# Emotional Processing in Healthy Ageing, Mild Cognitive Impairment, and Alzheimer’s Disease

**DOI:** 10.3390/ijerph18052770

**Published:** 2021-03-09

**Authors:** José Cárdenas, María J. Blanca, Fernando Carvajal, Sandra Rubio, Carmen Pedraza

**Affiliations:** 1 Department of Developmental and Educational Psychology, Faculty of Psychology, University of Malaga, Campus Universitario de Teatinos, s/n, 29071 Malaga, Spain; cardenas@uma.es; 2 Department of Psychobiology and Methodology of Behavioral Science, Faculty of Psychology, University of Malaga, Campus Universitario de Teatinos, s/n, 29071 Malaga, Spain; blamen@uma.es; 3 Department of Biological Psychology and Health, Faculty of Psychology, Autonomous University of Madrid Calle Ivan Pavlov, 6, 28049 Madrid, Spain; fernando.carvajal@uam.es; 4 The Biomedical Research Institute of Málaga (IBIMA), 29010 Malaga, Spain

**Keywords:** emotional processing assessment, diagnosis, mild cognitive impairment, Alzheimer’s disease

## Abstract

Emotional processing, particularly facial expression recognition, is essential for social cognition, and dysfunction may be associated with poor cognitive health. In pathological ageing conditions, such as mild cognitive impairment (MCI) and Alzheimer’s disease (AD), in which cognitive impairments are present, disturbed emotional processing and difficulty with social interactions have been documented. However, it is unclear how pathological ageing affects emotional processing and human social behaviour. The aim of this study is to provide insight into how emotional processing is affected in MCI and AD and whether this capacity can constitute a differentiating factor allowing the preclinical diagnosis of both diseases. For this purpose, an ecological emotional battery adapted from five subsets of the Florida Affect Battery was used. Given that emotion may not be separated from cognition, the affect battery was divided into subtests according to cognitive demand, resulting in three blocks. Our results showed that individuals with MCI or AD had poorer performance on the emotional processing tasks, although with different patterns, than that of controls. Cognitive demand may be responsible for the execution patterns of different emotional processing tests. Tasks with moderate cognitive demand are the most sensitive for discriminating between two cognitive impairment entities. In summary, emotional processing tasks may aid in characterising the neurocognitive deficits in MCI or AD. Additionally, identifying these deficits may be useful for developing interventions that specifically target these emotional processing problems.

## 1. Introduction

Emotional processing, particularly facial expression recognition, is essential for social cognition with clear and direct involvement in adequate human social behaviour [1]. Difficulties in processing emotional signals might have important implications for social interactions throughout life and are associated with low social competence and a poor social life ([2], reviewed in ref. [3]). Having a range of good social connections has been identified as an important aspect of successful ageing [4]. Conversely, impaired emotional processing is associated with specific reductions in social competence and interpersonal functioning [5,6,7], which may lead to social isolation and psychopathological disorders [8]. However, emotion may not be separated from cognition [9]. In fact, social isolation has been associated with deficient cognitive health [10,11].

In pathological ageing conditions, such as mild cognitive impairment (MCI) and Alzheimer’s disease (AD), in which cognitive impairments are present [12,13,14], disturbed emotional processing and difficulty with social interactions have been documented [15,16]. Neuropsychiatric and behavioural symptoms such as agitation, wandering, and aggression have been linked to impaired emotional processing, particularly to deficits in the ability to perceive and recognise the emotions of others. These behavioural impairments are common in patients with Alzheimer’s dementia [8]. However, it is unclear how pathological ageing affects emotional processing and human social behaviour. In this sense, it has been reported that AD patients have more severe impairments in emotion perception than patients with MCI and healthy older adults [16,17,18,19], while other studies show that people with AD have no deficits in emotional perception compared to controls [20,21,22].

Furthermore, the emotional processing deficit in MCI is very controversial [23,24,25]. MCI refers to a state of cognitive function that is abnormal for a person’s age and education level but does not meet criteria for clinically probable AD. However, some individual diagnosis of MCI may progress to AD [14,26,27]. Although not entirely conclusive, it has been suggested that facial emotion processing can be impaired in MCI prior to more marked cognitive deficits [20]. Most of the studies aimed at the neuropsychological characterisation of these conditions have focused on cognitive deficits, nevertheless, an emotional processing assessment may enable a more comprehensive and in-depth knowledge of the impairment profile in both MCI and AD. Understanding the first signs of emotional processing deficits is of great clinical significance because it specifically allows different strategies for prevention and intervention. In addition, it would be of great interest to be able to detect discrete cognitive impairment through some type of neuropsychological evaluation in order to start treatment as soon as possible, but no test with a high predictive value for the development of AD has been described so far.

The aim of this study is to provide further insight into how emotional processing is affected in MCI and AD compared to senior healthy controls. To this end, we used an ecological emotional battery adapted from five subsets of the Florida Affect Battery (FAB) [28,29,30,31]. This battery enables the assessment of emotional processing in different sensory modalities, including prosody and facial discrimination. This type of neuropsychological test would be a good predictive tool, both because of its low economic cost and its usefulness in routine clinical practice since it is easy to apply and is not influenced by one’s academic level. According to this idea, in a previous investigation we found that the decline in the task of categorising facial expression is more evident in a subgroup of Parkinson’s patients with greater global impairment (motor and cognitive) [32].

Conversely, cognitive demands can influence the execution of different emotional processing tests, particularly mnemonic and linguistic functions. In this sense, semantic memory may be impaired in clinically recognized states of cognitive impairment such as MCI and AD [33]. Moreover, language performance deficits can appear early before impairment in episodic memory, visuospatial construction ability, or mental status in individuals at risk for MCI [34]. In the same sense, AD is associated with a loss of semantic knowledge [35] and grammatical comprehension [36]. For this reason, the affect battery was divided into subtests according to cognitive demand that resulted in three blocks.

According to the abovementioned evidence, we expected that patients exhibiting either cognitive impairment entity, MCI or AD, would display emotional processing difficulties at varying levels of intensity. Thus, while participants with MCI would exhibit a slight but noticeable and measurable decline in emotional processing, AD participants would have a clear impairment. These deficits will be more evident when emotional tasks that require high memory and language load are performed.

## 2. Material and Methods

### 2.1. Sample

The sample comprised 101 individuals (33 males and 68 females) aged between 67 and 95 years (*M* = 81.93, *SD* = 6.28). A total of 45 were healthy control elderly individuals, 24 were patients with MCI, and 32 were patients with moderate AD. Participants were recruited from senior day centres, from nursing homes, via family caregivers of people with Alzheimer’s disease or from their own homes within the provinces of Malaga, Cádiz, Madrid and Valencia (Spain). All participants were able to follow instructions and understand the content of the assessment through verbal communications. Exclusion criteria included cases with disturbance of consciousness, delirium, psychiatry disorders, challenging behaviour, severe physical illness, and severe aphasia of a major sensorimotor impairment. The diagnosis of MCI was based on Petersen’s criteria (reviewed in ref. [37]), and a probable diagnosis of AD was based on the Diagnostic and Statistical Manual of Mental Disorders (4th ed.) (DSM-IV) and the National Institute of Neurological and Communicative Disorders and Stroke and the Alzheimer’s Disease and Related Disorders Association (NINCDS-ADRDA) criteria. All diagnoses were established by an experienced physician.

To describe and compare groups regarding age, depression, independence with respect to activities of daily living, and general cognitive status, we compared age and scores on the Geriatric Depression Scale (GDS-15, [38]), the Barthel index activities of daily living, and the Spanish version of the Mini-Mental Status Examination (MMSE-S, [39,40]). In the Spanish version, scores range from 0–35 instead of from 0–30. Scores higher than 30 indicate basically no cognitive impairment, scores between 25 and 29 indicate mild cognitive impairment, and scores less than 24 indicate cognitive impairment. The results are shown in Table 1. No differences among groups were observed in age, GDS-15 scores, or Barthel index scores. However, differences among groups on MMSE-S scores support higher cognitive impairment in the AD group, mild cognitive impairment in the MCI group, and no cognitive impairment in the control group. In fact, scores from all participants from the control group on the MMSE-S indicated no impairment.

### 2.2. Procedure

The study protocol was carried out following the rules of the Declaration of Helsinki of 1975 and approved by the Research Ethics Committee of Malaga University (CEUMA: 2014-0005-H). All participants or legally authorised representatives provided informed consent according to the declaration of Helsinki and the Spanish law on personal data protection (RD 1720/2007 and Organic Law 3/2018, of 5 December, on Data Protection and Guarantee of Digital Rights).

The research team contacted different senior day centres, nursing homes, and associations for families of people with Alzheimer’s and other dementias in the province of Malaga, Cádiz, Madrid, and Valencia (Spain) and informed them about the study’s objectives and procedures. Individuals from the institutions and associations that agreed to participate were invited to take part in the study, and those who agreed to participate completed the questionnaires (MMSE-S, GDS-15, Barthel index, and FAB). The tests were administered in two or three different sessions, lasting approximately one hour, separated by between 1 and 3 days, in the same room, for every subject. The number of sessions depended on the physical and/or cognitive status of the participants to avoid the possibility that fatigue could bias the results. The cognitive evaluation was carried out by experienced psychologists belonging to the staff of each of the centres and following a standardised protocol.

After completing the MMSE-S, GDS-15 and Barthel index tests, each subject individually performed a computerised battery of 13 tasks (for a more detailed description, please see Carvajal et al. 2007 and 2009). For this purpose, participants were seated at approximately 50 cm from a 17.3 in. 1.920 × 1.080-pixel computer screen. The order of presentation of tasks was counterbalanced. All the tests of emotional battery were administered by a trainer psychologist (JC).

### 2.3. Instruments

For the emotional processing assessment, all participants performed a computerised battery of tests composed of 13 tasks that require discrimination, recognition, and recall of facial emotions. These tasks, which have been used in several clinical studies, were adapted [28,29,30,31,32] from 5 subsets of the FAB [41]. This version is shorter than the original battery (which makes it easier to apply to a population with cognitive impairment). Different from the original, it includes a block of immediate and deferred memory tasks for faces, emotional facial expressions, and verbal emotional memory [31], which can be especially useful for evaluating pathologies affecting memory [29,30,31]. It also uses a discrimination task based on the paradigm «a face-in-the-crowd effect» [28], and the prosody test has been adapted to the Spanish-speaking population [30,42].

The battery was divided into three blocks according to cognitive demand. Tasks grouped in the first block included discrimination and facial emotion recognition tasks without the involvement of relevant grammatical and semantic aspect language. Tasks grouped in the second block included discrimination and facial emotion recognition tasks with the involvement of relevant grammatical and semantic aspects, including denomination, the classification and valence of facial emotional expression, and prosody identification. Finally, the tasks grouped in the third block included tasks of declarative memory, i.e., immediate and deferred memory for emotion-laden words and for emotional facial expressions and two recognition memory tasks of face identity and emotion. Table 2 shows a more detailed description of the tasks of each block.

### 2.4. Statistical Analysis

To examine emotional differences among groups (control, MCI, and AD groups), a series of one-way analysis of covariance with age as covariate (ANCOVA) and analyses of variance (ANOVA) was performed for the score on each task associated with emotional processing. The only statistically significant correlation found was between age and the score on the emotional labelling task, therefore, we performed an ANCOVA only for this variable. Since ANOVA is robust against non-normality [43], we focused on the homogeneity assumption. We followed the guideline proposed by Blanca et al. (2018) [44] regarding the control of Type I errors with respect to heterogeneity of variance with an unbalanced design. This guideline considers the variance ratio, the coefficient of group size variation, and the pairing between variance and group size. Measures of association size were reflected in eta squared (*η*^2^) values. The analyses were followed by multiple comparisons between groups with Bonferroni adjustment when required.

## 3. Results

Ten participants showed a missing value in at least one of the dependent variables. The Little’s test of missing completely at random did not reach statistical significance, χ^2^ (93) = 77.23; *p* = 0.88, indicating that missing values were randomly distributed across all observations. Therefore, we proceeded with the analysis using completed cases for each variable. The results from the ANCOVA and ANOVA series are shown in Table 3, Table 4 and Table 5. According to Blanca et al. (2018), in an unbalanced design, ANOVA controls Type I errors when the variance ratio is equal to or less than 1.5. This was the case for six of the 13 dependent variables. Of these remaining cases, five variables showed variance ratios between 1.6 and 3, but the pairing between variance and group size supported ANOVA robustness. Finally, 2 cases showed variance ratios between 4 and 6, but again, the pairing between variance and group size, considering the coefficient of group size variation, ensured the control of Type I errors. Based on these results, we considered it appropriate to proceed with the interpretation of the results from the statistical analysis.

### 3.1. Emotional Discrimination That Does Not Require the Involvement of Relevant Grammatical and Semantic Aspects of Language (Block 1)

Regarding the matrices task, the results revealed significant differences among groups in face and emotional facial discrimination task performance using the paradigm “a face-in-the-crowd effect” proposed by Hansen and Hansen (1988) [45]. Post hoc analysis indicated that AD participants exhibited more problems and had less accuracy in finding faces based on identity and/or facial expressions than older people without cognitive deficits. However, there were no differences between the control and MCI groups.

In the facial discrimination task (subtest 1 of the FAB), significant differences were observed among groups, showing that individuals with AD had difficulty discriminating facial identity compared to the performance by individuals in the MCI and control groups. Again, the results from individuals with MCI did not differ from those of the control group. Conversely, ANOVA results indicated that cognitive impairment did not affect performance on the facial affect discrimination task (subtest 2 of the FAB) (please see Table 3).

### 3.2. Emotional Discrimination That Requires the Involvement of Relevant Grammatical and Semantic Aspects of Language (Block 2)

The results revealed no differences among groups regarding the facial affect naming task (subset 3 of the FAB) and the emotional labelling task. There were differences in the Prosody task performance, showing that the AD group experienced difficulties on both tasks compared to the results of the control group.

Finally, in relation to the facial affect selection task (subtest 4 of the FAB), in which the participant had to select the photographs with the displayed emotion indicated by the experimenter, and significant differences were observed among groups, revealing significant differences between controls and individuals exhibiting either MCI or AD (please see Table 4).

### 3.3. Tasks That Involved a High Load Declarative Memory (Block 3)

Overall, the results support a decline in recognition memory in MCI and AD. Statistical analysis revealed that the average recognition error rates for the facial identity recall task (subtest 5A of the FAB) were greater for subjects with cognitive impairments (MCI and AD). Similarly, participants with cognitive impairments made significantly more errors than the control group on the recall of face emotions task (subtest 5B of the FAB).

Regarding emotional verbal memory, the results indicated significant differences among groups in scores on the immediate emotional verbal memory task (verbal S-T), showing that the AD and MCI groups had lower scores than the control group. Differences were also found in scores on the deferred emotional verbal memory task (verbal L-T) but only between the AD and control groups.

Finally, in relation to memory for facial emotional expression, the results showed that the immediate facial emotional expression memory task (facial S-T) was only impaired in AD. Nevertheless, the deferred facial emotional expression memory task (facial L-T) results differed in both the MCI and AD groups compared to those of the control group (please see Table 5).

## 4. Discussion

Determining sensitive assessment tools for detecting neuropsychological changes is crucial for the early identification of MCI and AD. Most research has focused on assessing cognitive functions but evaluating emotional processing may also be a sensitive way to achieve a better characterisation of the clinical profile of both entities, which may entail better implementation of effective strategies for slowing the decline of cognitive and social abilities [19]. Hence, this study was designed to examine how emotional processing is affected in MCI and AD. For this purpose, we used an ecological emotional battery adapted from five subsets of the Florida Affect Battery (FAB). Given that emotional processing may be influenced by concurrent cognitive demand [46], the battery tasks were grouped into three blocks. Cardinal MCI and AD symptoms are memory impairments [33,47] and linguistic function decline [34,35]. For this reason, the tasks were grouped considering whether semantic and grammatical, mnemonic, or none of these cognitive functions were directly and mainly involved. This battery, in addition to being ecological and adapted to the Spanish-speaking population, is composed of a wide range of tasks including not only face recognition and discrimination tests and emotional labelling, but also valence, prosody, and memory tests for facial expressions. This wide range of tasks can help to better characterise emotional disturbances in older populations with cognitive impairment such as AD and MCI.

In the emotional battery, considering results from the discrimination task that do not require a clear involvement of grammatical and semantic aspects of language, the AD group showed poorer performance on the discrimination of facial identity (facial discrimination task). Nevertheless, the ability to discriminate facial emotions was relatively preserved depending on the complexity of the task. Similar to what has been observed by other authors, AD participants had difficulty discriminating facial emotions when a complex task that assessed “a face-in-the-crowd effect” (matrix) was used [15], but not with a simpler facial affect discrimination task that required the participant to indicate whether a pair of faces depicted the same or a different emotion [15,19]. These results differ from those found using the same version of the affect battery in patients with degenerative disease (dementia associated with Parkinson’s disease). These data would reveal that possibly different neurobiological substrates may be responsible for these effects. Thus, the problems found in facial expression recognition may be associated with the progressive neuronal loss in frontostriatal and mesolimbic circuits, which characterises Parkinson disease [30]. However, the MCI group did not show any impairment in facial and emotional discrimination. Together, these results revealed that in AD patients, facilitation elicited by emotional content was observed when a simple discrimination task was used. Nevertheless, this population showed problems with discrimination identity, and partially, facial emotions, despite the tasks not requiring a high memory load or semantic and grammatical aspects of language. Deficits in visual tasks are also commonly reported in AD [48]. However, impairment in discrimination tasks may not be attributed unequivocally to perceptual processes. In fact, AD patients were partially able to distinguish between different static facial expressions when the complexity of the task was reduced. Matching tasks such as matrices and subtest 1 of the FAB used involve many processes (attention, motor response, decision making, among others), contributing to performance [49]. Given that AD patients usually have poor general cognitive functioning beyond linguistic or mnemotechnic difficulties, cognitive problems such as attention, decision making, or working memory may explain this deficit, at least in part, because emotional facilitation of face discrimination was not observed during the complex affect discrimination task. These alterations observed in AD patients that were not seen in MCI may be due, at least in part, to greater temporal lobe degenerative involvement. In this sense, with the aim of deepening the understanding of this circuitry underlying emotional processing we have also carried out an extensive study with patients with temporal lobe epilepsy with unilateral resection of the hippocampus and amygdala [28,29]. Our findings indicate that there is a dissociation between processing facial identity and facial expression, and a clear involvement of the temporal lobe and a degree of functional and hemispherical specialisation. Collectively, AD participants, unlike controls or MCI participants, had problems with discrimination tasks, and emotional facilitation was observed in a simple facial emotion discrimination task.

Then, the implications of language’s role in emotional processing were also explored. Participants were asked to match a range of facial emotional expressions with the name (or value) of the presented valence and intensity (facial affect naming task, subtest 3 of the FAB). It has been considered that an approach based on the intensity of emotions can be more sensitive to detecting subtle deficiencies in facial emotion recognition. However, very few studies take into account the intensity of emotions [50]. In consonance with prior studies indicating intact emotional ratings in AD [51,52,53], the results revealed an unimpaired capacity to match the emotional valence or intensity of facial expression with the name presented. Both MCI and AD participants performed these tasks at the same level as controls in rating the emotional descriptions for valence and arousal. Therefore, the intensity of facial expressions has not helped to discriminate between clinical populations. Alterations in mood could be a factor affecting results, but this possibility has to be ruled out as no differences between the groups were observed in the GDS-15 test. However, regarding language skills, different from what Bucks and Radford (2004) found with prosody tasks of Florida Affect Battery [19], AD participants have problems related to understanding prosody, exhibiting difficulty in prosody-emotional sentence matching tasks. Cultural differences may explain, at least partially, the discrepancies between studies. However, it has been described that processing information relating to affective tone and impaired prosody comprehension impairments occur early in the disease course and remain stable as cognitive function declines [36]. This task has been particularly sensitive to damage to the left hippocampus. Patients with left temporal lobectomy showed impairment in discriminating facial expressions, in the memory of a facial expression, and/or in naming facial expressions [29]. In HIV+ individuals, a strong association between lower left hippocampus volumes and poorer test scores was found [30], which may be due to the demands of the task of matching emotional prosody to visual faces. Affective prosody is a powerful signal for social communication therefore, loss of affective-prosodic comprehension may have an impact on social relationships [54]. Furthermore, AD participants performed worse than the rest of the groups on emotional labelling but did not reach statistical significance. Anomia is one of the most evident linguistic symptoms in AD, beginning in the initial phase of the disease and becoming particularly pronounced on visual confrontation naming tasks [55], including emotional labelling [24]. Anomia may be related to the inability of a patient to access the phonological label for a particular word, and over the course of the disease, it may reflect a semantic knowledge disruption. It has been documented that impaired access to the meaning of emotion-related words impairs the ability to perceive emotions on faces [56], which may explain, at least partially, emotional discrimination problems observed in AD. However, problems to remember names, things, and places are a frequent complaint of older people and they often fail to form association between faces and names or to recall names from faces [57]. Thus, during this period of life, difficulties can be observed between the association of a name and a face (based on facial identity) [58,59]. Older people are also less accurate in labelling the facial expressions of some emotions [60]. It could therefore be that they also have difficulty in associating an emotional facial expression with the corresponding name. These difficulties could also explain why, although there is a clear deterioration in Alzheimer’s, no differences have been observed between the different groups. This could be investigated in the future. On the other hand, AD participants did not have problems selecting a verbal category from a given facial expression. Moreover, by using naming tests, subjects with AD benefited from phonemic cues [61] suggesting that the labelling and emotional facial discrimination difficulties observed in individuals with moderate AD may not be completely explained by semantic problems.

On the other hand, MCI and AD participants had difficulty selecting one of the five photographs that displayed the emotion indicated by the experimenter (facial affect selection task, subtest 4 of the FAB). Given that participants with MCI had not demonstrated problems in emotional labelling, other cognitive functions besides impaired language should not be ruled out. In subtest 4 of the FAB, beyond the ability to assign a name to a facial expression, attention and working memory, including decision making, with a clear involvement of language, are required. Both the MCI and AD groups displayed impaired attentional processing and working memory capacity [62,63,64,65], which may explain the observed deficits in performance on this task. Along the same lines, problems in facial expression recognition task of subtest 4 of the FAB, which have also been found in neurocognitively affected HIV+ patients, but not in patients without cognitive impairment [30]. Together, when there is clear language involvement in emotional processing, emotional labelling and prosody comprehension may be more useful to distinguish between MCI and AD neuropsychological impairments. Otherwise, the simplest (FAB3) or complex task (FAB4) is not effective in discriminating between both entities.

Finally, the performance on emotional processing with a high explicit memory demand was evaluated. For this, facial identity and emotion recall (subtest 5A and 5B of the FAB, respectively), immediate and deferred verbal memory (for emotion-laden words), and immediate and deferred facial emotional expression memory were assessed. Emotional content enhanced recognition [66] and strength memory traces for emotion-laden information in normal subjects, whereas for Alzheimer participants, emotions did not benefit memory. As expected, AD participants exhibited clear memory problems. Memory performance was the worst in the AD group irrespective of the tasks and, consistent with most laboratory evidence, participants with AD were unable to experience declarative memory enhancement via emotional information [66,67,68,69]. MCI patients had poor memory overall. Our results suggest that the effects of emotion on memory were not completely preserved in patients with MCI. Thus, immediate memory performance for emotional facial expressions was similar to that observed in the control group and that of immediate and deferred emotional verbal memory was situated on the spectrum between healthy ageing and AD individuals, while performance on identity and emotional information recall of faces and deferred memory for emotional facial expressions was similar to that seen in AD participants. Together, we observed that despite poor memory accuracy overall in MCI patients, there were differences in the pattern of memory impairment with respect to AD.

This study has certain limitations that must be acknowledged. First, the use of a cross-sectional design means that no causal relationships can be inferred from the results. Second, we recruited participants by means of convenience sampling, thus restricting the generalisability of the findings. Thirdly, given the sample size, control for certain sociodemographic variables (e.g., sex, education) has not been included in the statistical analysis. Fourth, the group of patients with mild cognitive impairment was not been divided by subtypes. It would be interesting in future studies to include this information in order to analyse its impact on emotional processing. Despite these limitations, the study makes an important contribution. Thus, our data reveal that measuring emotional processing with tasks that require different cognitive demands can help to better characterise deterioration in DLB and AD. Our results showed poorer performance by the AD group compared with that of the control group on the emotional processing tasks, except for a simple emotional discrimination task or a task of assigning the valence and intensity of facial expressions. Nevertheless, in the MCI group, the pattern of impairment depends on the difficulty of the task and cognitive demand. MCI participants performed at the same level as the control group in discrimination tasks, emotional labelling, prosody comprehension tasks, and immediate facial emotional memory tasks and performed similarly to mild AD patients when the tasks were more complex or required the consolidation of information. The observed difficulties may not be explained by depression symptoms, as no differences in GDS-15 scores were observed among groups. As expected, MCI participants had more difficulties in tasks that required attentional processing, decision making, and working memory capacity with a clear involvement of language or tasks with a high declarative memory load. AD participants showed difficulties in almost all tasks regardless of whether a high memory or language load was required.

The emotional battery employed in our study takes into account the multimodal nature of human social relationships involving the processing of visual facial cues, the tone of voice, the choice of words, and the memory for facial identity and emotions. In addition, the reduced length of tasks makes it more appropriate for assessing patients with cognitive impairment. Although emotional processing may be influenced by concurrent cognitive demand, social cognition changes are not entirely dependent on the cognitive level and increase over time [70]. For this reason, identifying social deficits with an ecological tool, combined with other neuropsychological tests, can be useful as an early diagnostic test [71]. The emotional battery may improve the predictive ability of an AD risk score, combined with other neuropsychological test scores.

Thus, these patients can benefit from a possible pharmacological and neuropsychological intervention that modifies the evolutionary course of the disease. In this sense, in AD patients, combined emotional rehabilitation and cognitive stimulation induced significant improvement not only on the recognition of facial emotions, but also on processing speed, basic activities of daily living, and scores on the Folstein Minimental Test [72].

## 5. Conclusions

Here, we used an ecological emotional battery adapted from five subsets of the Florida Affect Battery. The division of this battery into three blocks according to cognitive demand revealed that MCI participants had more difficulties in tasks that required attentional processing, decision making, and working memory capacity with a clear involvement of language or tasks with a high declarative memory load. AD participants showed difficulties in almost all tasks regardless of whether a high memory or language load was required. Identifying emotional deficits may aid in characterising the neurocognitive deficits in MCI or AD and may be useful for developing interventions that specifically target these emotional processing problems.

## Figures and Tables

**Table 1:** Characteristics of the control, mild cognitive impairment (MCI) and Alzheimer’s disease (AD) groups according to age and scores on Geriatric Depression Scale (GDS-15), the Barthel index activities of daily living, and the Spanish version of the Mini-Mental Status Examination (MMSE).

Variables	Control	MCI	AD	*F*	Comparisons
	*M (SD)*	*M (SD)*	*M (SD)*		
Age	80.76 (6.59)	83.00 (5.58)	82.78 (5.42)	1.44	
GDS-15	3.46 (2.70)	4.38 (3.06)	4.16 (2.09)	1.19	
Barthel Index	77.78 (28.78)	75 (26.94)	68.13 (29.69)	1.08	
MMSE-S	32.49 (1.53)	27.17 (1.58)	18.72 (3.15)	370.75 ***	C vs. MCI; C vs. AD; MCI vs. AD

Note. *** *p* < 0.001.

**Table 2:** Tasks which compose the ecological emotional battery adapted from five subsets of the Florida Affect Battery (FAB).

Block	Task	Description
Block 1. Discrimination tasks	Matrices	Facial identity and emotional expression discrimination in a task using the paradigm «a face-in-the-crowd effect» Range of score: 0–60
	Facial Discrimination Task (subtest 1 of the FAB)	Subjects had to decide whether the photographs were of the same person or not. Range of score: 0–10
	Facial Affect Discrimination Task (subtest 2 of the FAB)	Participants had to decide whether the faces showed the same or different emotional expressions. Range of score: 0–15
Block 2. Discrimination and selection tasks (with a clear language participation)	Facial Affect Naming Task (subtest 3 of the FAB)	Participants had to choose from emotional word categories (and intensity) that best corresponded to the expression in the photograph. Range of score:0–15
	Emotional Labelling	Participants had to labelling emotional facial expressions. Range of score: 0–15
	Facial Affect Selection Task (subtest 4 of the FAB)	From five photographs of each screen, the participants had to select which of the five photographs displayed the indicated emotion. Range of score: 0–10
	Prosody	Participants had to decide if there is affective congruence between sound and meaning of sentences. Range of score: 0–9
Block 3. Memory tasks.	Recall of face identity (subtest 5 A of the FAB)	Participants had to identify the photograph that showed the identity they had seen before, regardless of the facial expression of the model. Range of score: 0–10
	Recall of face emotion (subtest 5 B of the FAB)	Participants had to identify the photograph that showed the same facial expression they had seen before, regardless of the identity of the model. Range of score: 0–10
	Immediate emotional verbal memory (Verbal S-T)	Participants had to remember which emotional word was associated with a neutral word, immediately after its presentation. Range of score: 0–18
	Deferred emotional verbal memory (Verbal L-T)	Participants had to remember which emotional word was associated with a neutral word, 10 m after its presentation. Range of score: 0–6
	Immediate facial emotional expression memory (Facial S-T)	Among 18 pictures, participants had to choose which six had been seen before (same emotion and identity), immediately after its presentation. Range of score: 0–18
	Deferred facial emotional expression memory (Facial L-T)	Among 18 pictures, participants had to choose which six had been seen before (same emotion and identity), 10 m after its presentation. Range of score: 0–6

Note: Verbal S-T = verbal short-term memory; verbal L-T = verbal long-term memory; facial S–T = short-term memory for facial emotional expressions; and facial L-T = long-term memory for facial emotional expressions.

**Table 3:** Emotional processing in tasks that do not require a relevant involvement of grammatical and semantic aspects of language. Mean (*M*) and standard deviation (*SD*) of the dependent variables according to diagnosis, and *F*-statistics from ANOVA and eta squared.

Variables	Control	MCI	AD	*F*	*p*	*η* ^2^	Comparisons
	*M (SD)*	*M (SD)*	*M (SD)*				
Matrices	41.82 (9.88)	37.7 (11.22)	34.80 (11.22)	4.06	0.02	0.08	C vs. AD
FAB 1	9.04 (1.40)	8.96 (1.40)	7.75 (1.72)	7.71	<0.01	0.14	C vs. AD; MCI vs. AD
FAB 2	11.36 (1.78)	10.46 (2.81)	10.84 (1.93)	1.52	0.22	0.03	

Note. *n* (control, MCI, AD): Matrices: 45, 24, 30; FAB 1: 45, 24, 32; FAB 2: 44, 24, 32.

**Table 4:** Emotional processing in tasks that require a relevant involvement of grammatical and semantic aspects of language. Mean (*M*) and standard deviation (*SD*) of the dependent variables according to diagnosis, and *F*-statistics from ANOVA and eta squared.

Variables	Control	MCI	AD	*F*	*p*	*η* ^2^	Comparisons
	*M (SD)*	*M (SD)*	*M (SD)*				
FAB 3	9.38 (2.41)	9.30 (2.18)	8.20 (2.48)	2.44	0.09	0.05	
Labelling	8.62 (2.14)	8.67 (2.75)	7.51 (1.87)	3.25	0.06	0.06	
FAB 4	7.41 (1.64)	6.46 (1.66)	6.55 (1.63)	3.67	0.03	0.08	C vs. MCI; C vs. AD ^a^
Prosody	3.72 (1.81)	3.32 (1.46)	2.63 (1.35)	4.16	0.02	0.08	C vs. AD

Note. *n* (control, MCI, and AD): FAB 3: 45, 23, 30; Labelling: 45, 24, 30; FAB 4: 44, 24, 31; Prosody: 44, 22, 30. ^a^ one-tailed Bonferroni adjustment.

**Table 5:** Tasks that involved a high load of immediate and deferred memory for emotion-laden words and for emotional facial expressions. Mean (*M*) and standard deviation (*SD*) of the dependent variables according to diagnosis, F-statistics from ANOVA and eta squared.

Variables	Control	MCI	AD	*F*	*p*	*η* ^2^	Comparisons
	*M (SD)*	*M (SD)*	*M (SD)*				
FAB 5 A	7.18 (2.09)	5.79 (1.95)	5.50 (2.20)	6.82	<0.01	0.12	C vs. MCI; C vs. AD
FAB 5 B	4.58 (1.98)	3.50 (1.91)	3.40 (1.32)	4.93	<0.01	0.09	C vs. AD
Verbal S-T	3.82 (3.30)	2.13 (2.62)	0.84 (1.60)	11.20	<0.01	0.19	C vs. MCI; C vs. AD
Verbal L-T	1.11 (1.31)	0.58 (0.76)	0.15 (0.51)	8.63	<0.01	0.15	C vs. AD
Facial S-T	9.42 (2.92)	8.04 (3.27)	5.72 (3.54)	12.41	<0.01	0.20	C vs. AD; MCI vs. AD
Facial L-T	3.36 (1.41)	2.21 (1.25)	1.90 (1.62)	10.49	<0.01	0.18	C vs. MCI; C vs. AD

Note. *n* (control, MCI, and AD): FAB 5 A, B: 45, 24, 30; Verbal S-T, L-T, and Facial S-T: 45, 24, 32; Facial L-T: 44, 24, 30.

## Data Availability

The data presented in this study are available on request from the corresponding authors due to ethical considerations.
